# All-optical 2D perovskite photodetector arrays for pixelated long-wave infrared imaging

**DOI:** 10.1038/s41377-026-02442-y

**Published:** 2026-11-02

**Authors:** Yanyan Li, Zheng Qian, Du Chen, Conrad A. Kocoj, Shunran Li, Renee Wu, Xiang Shi, Ralu Divan, David A. Czaplewski, Benjamin T. Diroll, Ming Lu, Yugang Zhang, Shu Hu, Peijun Guo

**Affiliations:** 1 Department of Chemical and Environmental Engineering, Yale University, New Haven, CT USA; 2 Energy Sciences Institute, Yale University, West Haven, CT USA; 3 Center for Nanoscale Materials, Argonne National Laboratory, Lemont, IL USA; 4 Center for Functional Nanomaterials, Brookhaven National Laboratory, Upton, NY USA

**Keywords:** Optical materials and structures, Imaging and sensing

## Abstract

Infrared detection and imaging are important for applications including thermal monitoring, night vision, environmental sensing, and molecular identification. All-optical mid-wave infrared (MWIR) and long-wave infrared (LWIR) detection offers a compelling alternative to conventional infrared detectors by enabling non-contact photothermal sensing without needing electronic readout circuitry. Here, we demonstrate pixelated LWIR imaging using solution-processed two-dimensional metal-halide perovskites (2D-MHPs), whose strong electron-phonon interaction produces a large temperature coefficient of reflectance in the visible wavelength range. By integrating (PEA)_2_PbI_4_ films with LWIR-absorbing SiNₓ membranes, we realize centimeter-scale imagers capable of detecting 9.5-µm LWIR light with sensitivity down to ~20 pW·µm⁻². To mitigate lateral thermal diffusion that limits spatial fidelity, we introduce a structured-probe illumination scheme, and we further develop a fully pixelated imager with thermally isolated membrane-disc arrays. This design effectively eliminates pixel-to-pixel crosstalk and enables high-resolution optical readout using a silicon-based CMOS camera. These advances establish 2D-MHP-based all-optical thermal imaging as a scalable, low-cost platform for LWIR sensing, with strong potential for extension to far-infrared and terahertz detection.

## Introduction

Detecting infrared radiation is critically important because a vast amount of physical, chemical, and biological information is encoded in the infrared spectrum. Thermal emission in the mid-wave infrared (MWIR) and long-wave infrared (LWIR) provides a direct, non-contact window into the temperature, heat flow, and energy dissipation in materials and devices, enabling applications ranging from thermal and biomedical imaging to process monitoring and environmental sensing^1,2^. At the molecular scale, organic chemical species exhibit characteristic vibrational resonances in the infrared, making infrared detection important for chemical identification and medical diagnostics^3–5^. In addition, infrared photodetection underpins night vision, autonomous navigation, and semiconductor inspection, where the ability to resolve faint thermal signatures or weak vibrational fingerprints is crucial^6–9^. For these reasons, new materials and concepts are highly desirable for compact, low-power, and scalable infrared detectors that can operate across wide infrared spectral regimes and at ambient temperature.

In recent years, several emerging material platforms have been explored for infrared photodetection, including colloidal nanocrystals^10^, quasi-one-dimensional excitonic insulator systems^11^, two-dimensional (2D) layered materials such as graphene and black phosphorus^12,13^, and charge-density wave systems^14^. In these demonstrations, infrared light either excites charge carriers to generate a measurable photovoltage or photocurrent^15,16^, or their energy is converted into heat that raises the material temperature, which is read out through changes in electrical resistivity, as in bolometer detectors^17,18^. Leveraging the distinctive light-matter interactions and strong field effects present in some of these materials, additional novel concepts have also emerged, such as infrared detection mediated by strong coupling between molecular vibrations and tip-enhanced plasmonic near fields^19^, ratiometric infrared sensing involving transitions between different 4 f orbitals in rare-earth containing nanomaterials^20^, as well as single-detector spectrometers based on electrical gating and tunable moiré quantum geometry in 2D materials^21,22^.

Despite these advances, most demonstrations based on emerging materials remain limited to single-channel detection, where only the overall intensity of the incident infrared radiation is measured. Scaling such concepts into multipixel imaging arrays poses significant challenges, stemming from the difficulty of producing large-area, high-quality 1D and 2D materials and the complexity of fabricating dense, multiplexed electrical contacts. Developing materials and detection strategies that are intrinsically scalable and compatible with signal pixelation is crucial for realizing infrared imagers and cameras for high-quality infrared image acquisition.

2D metal-halide perovskites (2D-MHPs) have emerged as a versatile class of semiconductors with solution processability^23^, tunable bandgaps^24^, large exciton binding energy^25^, and strong light-matter interactions^26^. In particular, their pronounced exciton-phonon interactions lead to a high sensitivity of exciton linewidth to lattice temperature^27,28^. This property was recently exploited for mid-infrared photodetection at ambient conditions, where exciton-induced reflectance responds strongly to infrared-induced lattice heating, enabling an alternative bolometric mechanism^29^. Here, leveraging the 2D-MHPs, we demonstrate a scalable approach to pixelated imagers operating in the LWIR range. The LWIR imager operating in an all-optical scheme evolves from using centimeter-sized membrane, to the incorporation of structured probe illumination, and finally the use of pixelated membrane arrays. Compared to conventional photovoltaic and photoconductive infrared detectors, our all-optical detection offers an advantage in that it removes the requirement for complex readout integrated circuits (ROICs), thereby simplifying fabrication and lowering overall system cost. The detection efficiency demonstrated here down to the tens of pW·µm^-2^ can be further enhanced through cryogenic operation or by integrating optically resonant structures that amplify differential changes in optical reflectance. Our generalizable approach can be extended to detect electromagnetic waves across broader spectral regimes, from the far-infrared to the terahertz. Overall, this work paves the way toward compact, low-cost, and scalable infrared detectors based on earth-abundant materials—potentially enabling new opportunities for accessible, chip-integrated optical sensing technologies.

## Results

**Fig. 1:**
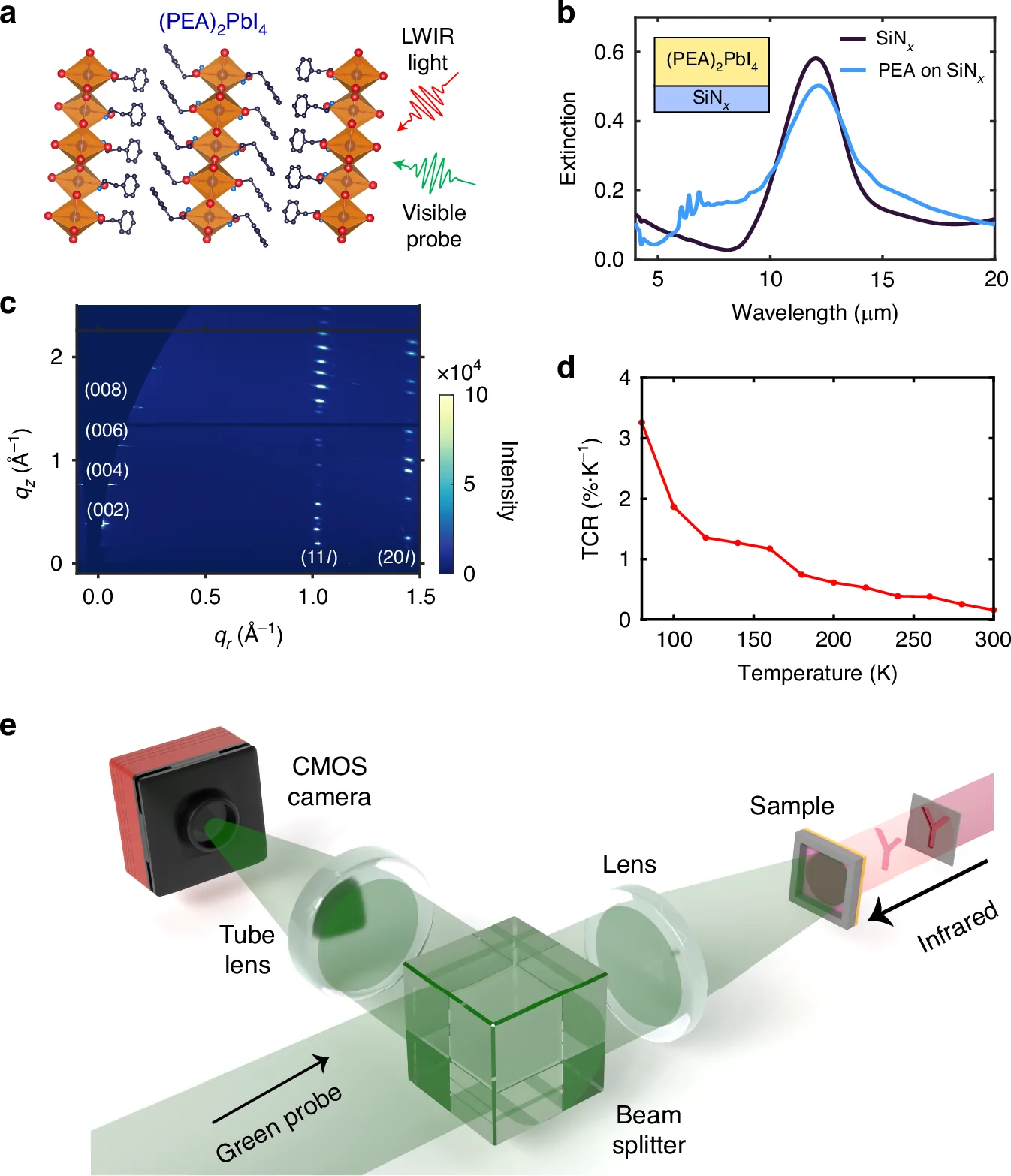
**a** Conceptual illustration of the all-optical detection of LWIR light with 2D-MHPs. **b** Extinction of infrared light by the vibrational resonance of SiN_*x*_ membrane with a thickness of 265 nm, both before and after the spin-coating of (PEA)_2_PbI_4_ film. **c** GIWAXS map for spin-coated (PEA)_2_PbI_4_ film showing a high degree of out-of-plane orientation. **d** Temperature coefficient of reflectance (TCR) plotted as a function of temperature. **e** Schematic drawing of the all-optical imaging of LWIR light using centimeter-sized (PEA)_2_PbI_4_/SiN_*x*_ membrane

The working principle of the all-optical infrared detection is conceptually depicted in Fig. 1a. The incident LWIR light, through a LWIR light absorber (*vide infra*), induces a lattice temperature rise in a 2D-MHP thin film, here (PEA)_2_PbI_4_, where PEA^+^ denotes the phenylethylammonium cation. The temperature rise causes a sensitive variation in the reflectance of (PEA)_2_PbI_4_ near the exciton resonance in the visible wavelength range (centered around 510 nm, corresponding to a wavelength in green). In essence, this all-optical detection scheme converts the detection of LWIR photons—typically challenging due to the small photon energy—into the detection of visible light, which can be efficiently measured using mature, cost-effective, and sensitive detectors such as photo-multiplier tubes (PMTs) or CMOS cameras. The LWIR is mechanically modulated by an optical chopper, a standard practice for other infrared detectors such as InSb and PbS, to enable comparison of visible-reflectance signals with LWIR-on and LWIR-off conditions.

Since the electronic bandgap of (PEA)_2_PbI_4_ and its absorption cut-off wavelength are in the visible range, (PEA)_2_PbI_4_ by itself is transparent to LWIR light. To enable LWIR absorption, we utilized a thin, low-stress silicon nitride (SiN_*x*_) membrane as the support layer for spin-coated (PEA)_2_PbI_4_, which is a scalable platform grown on Si wafers using low-pressure chemical vapor deposition. Low-stress SiN_*x*_ has high mechanical robustness and is commercially used for transmission electron microscopy membrane grids with thickness down to 20 nm. As shown in Fig. 1b, Si-N stretching vibration in a 265 nm-thick SiN_*x*_ membrane leads to pronounced LWIR vibrational absorption between 9 µm and 14 µm^30^. The peak extinction, approaching 60%, with a full width at half maximum of ~3 µm, is remarkably high considering the membrane thickness is only about 1/40 of the incident wavelength. The strong LWIR absorption coefficient reduces the thermal mass of the SiN_*x*_ membrane, in turn maximizing the temperature rise in the (PEA)_2_PbI_4_ film deposited on top, desirable for LWIR detection. The strong LWIR extinction by SiN_*x*_ is preserved after spin-coating a ~ 1 µm-thick (PEA)_2_PbI_4_ film on the SiN_*x*_ membrane (Fig. 1b). The slight variation in the 5 to 10 µm regime after the (PEA)_2_PbI_4_ coating likely arises from thin-film interference effects, given the relatively large thickness of the (PEA)_2_PbI_4_ (1.0 ~ 1.1 µm, Supplementary Fig. S3).

Grazing incidence wide-angle X-ray scattering (GIWAXS) data (Fig. 1c) confirms the preferential out-of-plane orientation of the (PEA)_2_PbI_4_ film, where the lead-iodide octahedral layers are aligned parallel to the SiN_*x*_ membrane. The temperature coefficient of reflectance (TCR), defined as the percentage change in reflectance per kelvin change in lattice temperature, was measured for the (PEA)_2_PbI_4_/SiN_*x*_ stack (Fig. 1d). Temperature-dependent absorbance and reflectance data are presented in Supplementary Fig. S2. Near the ambient temperature, a TCR of 0.2 ~ 0.3% is achieved around 510 nm, a wavelength near the exciton resonance where the sample has the strongest sensitivity to temperature. The out-of-plane crystallographic orientation of the (PEA)_2_PbI_4_ film is favorable as it reduces thermal dissipation rate and improves the TCR^25^. Although the TCR value increases by more than an order of magnitude when going from the ambient to liquid-nitrogen temperatures, suggesting dramatically enhanced sensitivity under cryogenic conditions, all subsequent experiments in this work were performed under ambient conditions (room temperature; relative humidity in the 30% ~ 50% range).

While our previous study demonstrated that a (PEA)_2_PbI_4_/SiN_*x*_ bilayer structure can function as an effective single-channel infrared detector^29^, the use of a PMT for measuring the visible probe reflectance is not scalable for imaging applications. To extend this detection concept to a full-field imaging modality, we implemented a CMOS camera-based detection scheme to capture the reflected green probe light, illustrated in Fig. 1e. In this configuration, the all-optical nature eliminates the need for electrical contacts, enabling direct photothermal imaging of the (PEA)_2_PbI_4_ film using a Si-based CMOS camera under monochromatic, continuous green probe illumination produced by a light-emitting diode. The LWIR light is incident from the (PEA)_2_PbI_4_ side of the bilayer stack, whereas the green probe illuminates the sample from the opposite side through the SiN_*x*_ membrane. Because SiN_*x*_ is transparent in the visible range, the probe light penetrates through the SiN_*x*_ membrane and interacts with the thermally sensitive (PEA)_2_PbI_4_ layer. The reflected probe light is then redirected by a beam splitter and collected by the CMOS camera.

Importantly, a spatially varying LWIR intensity profile, subject to detection, induces a corresponding inhomogeneous temperature rise across the (PEA)_2_PbI_4_/SiN_*x*_ stack, which is transduced into a reflectance change in the green that is directly mapped onto the CMOS camera sensor. Two imaging lenses are incorporated to ensure that the sample plane and CMOS sensor are optically conjugate, maintaining high-fidelity imaging across the entire field of view—which is determined by the CMOS sensor size.

In our proof-of-concept LWIR-detection measurements, LWIR radiation was produced by a quasi-continuous-wave quantum cascade laser (QCL) outputting monochromatic light with 9.5-µm wavelength. To demonstrate the imaging capability, the LWIR light was spatially structured using a metallic shadow mask, forming a logo “YALE CEE”, which was projected onto the (PEA)_2_PbI_4_/SiN_*x*_ membrane imager. The LWIR radiation was mechanically modulated at 50 Hz with an optical chopper. The CMOS camera, synchronized with the optical chopper, acquires images at a frame rate of 100 Hz (i.e. twice the chopping frequency). The differential reflectance signal was measured as $\triangle R/R=\left[{R}_{{{{\rm{on}}}}}-{R}_{{{{\rm{off}}}}}\right]/{R}_{{{{\rm{off}}}}}$, where ${R}_{{{{\rm{on}}}}}$ and ${R}_{{{{\rm{off}}}}}$ represent the spatially dependent sample reflectance with and without LWIR irradiation, respectively. The exposure time of the CMOS camera is approximately 80% of the duty cycle (i.e., 8 ms for the 10-ms duty cycle at a 100-Hz framerate). The measured spatial maps of △R/R serve as the all-optical photothermal signal across the imaging field of view.

**Fig. 2:**
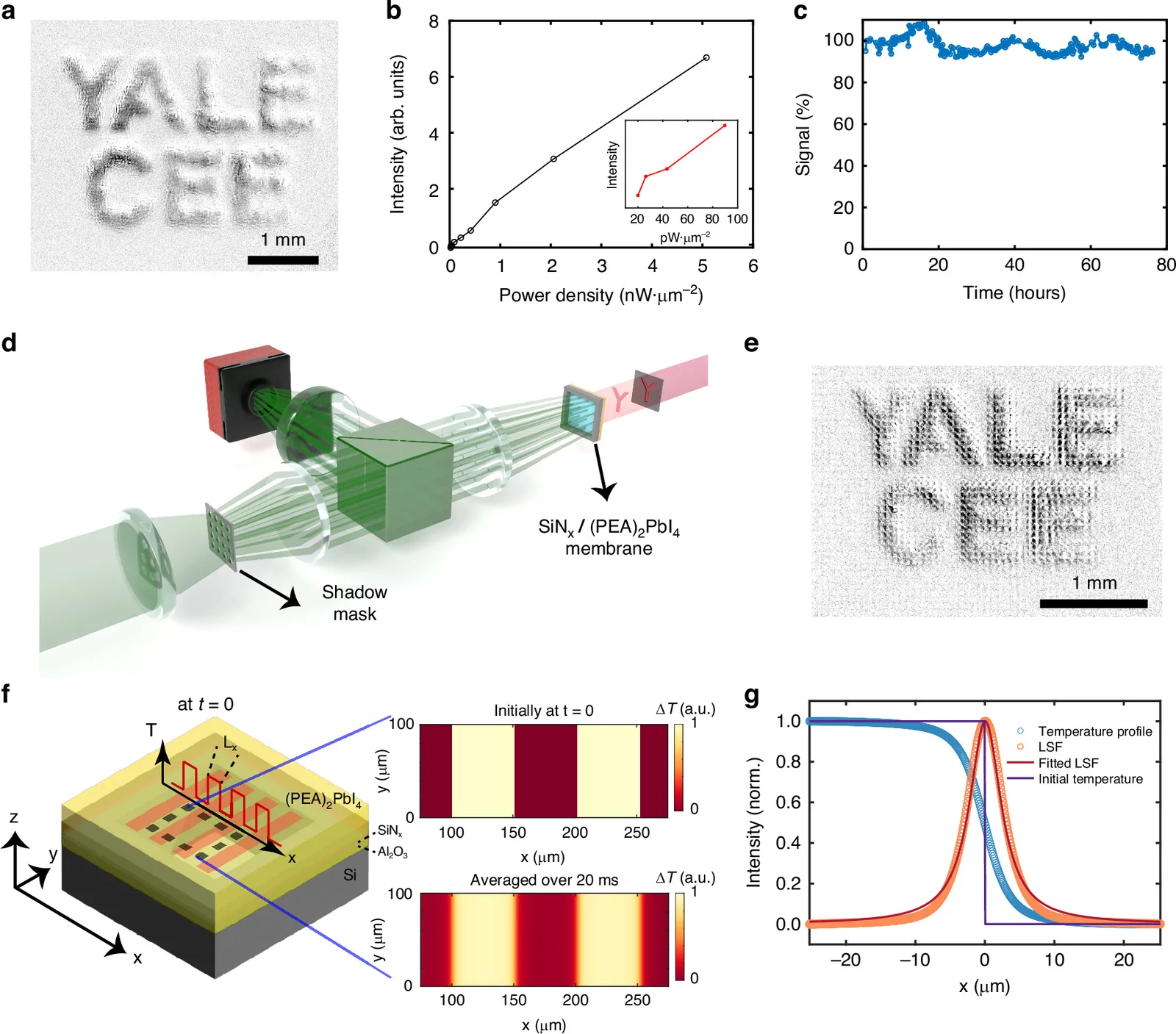
**a** All-optically detected image of a “YALE CEE” logo of LWIR light (9.5 µm wavelength) using (PEA)_2_PbI_4_/SiN_*x*_ membrane-based imager. The imaging contrast corresponds to △R/R induced by the LWIR irradiation, which is structured using a shadow mask as illustrated in Fig. 1e. **b** Measured intensity of △R/R as a function of incident power density of LWIR light. **c** △R/R intensity vs. irradiation time of LWIR light continuously measured over 80 hours. The power density of LWIR irradiation is 2.1 nW·µm^-2^. **d** Schematic illustration of structured visible probe beam for reducing crosstalk between mask-defined pixels on the (PEA)_2_PbI_4_/SiN_*x*_ membrane imager. **e** All-optically detected image of a “YALE CEE” logo of LWIR light (9.5 µm wavelength) using (PEA)_2_PbI_4_/SiN_*x*_ membrane-based imager and structured probe illumination. **f** FEM simulation of the thermal-diffusion-limited imaging performance of the membrane-based imager. The upper heat map shows the initial temperature distribution, and the lower heat map shows the temperature profile averaged over the first 20 ms. **g** The broadening of a sharp edge due to thermal diffusion. The solid purple line is the initial temperature distribution, and the blue circles are the temperature profile of the edge region averaged over the first 20 ms. The orange circle and solid line are the line spread functions (LSF) of the broadened edge, extracted from the derivative of the temperature profile and fitted with a Lorentzian distribution, respectively

Figure 2a presents a representative image from the LWIR-sensing measurements, clearly demonstrating LWIR imaging capability without the need for a ROIC. A uniform 5-mm field of view is obtained, reflecting the excellent film-forming uniformity of the 2D-MHP layer. To assess the sensitivity of the LWIR imager, we varied the power density of the incident LWIR radiation. As shown in Fig. 2b, the imager exhibits a desirable linear response over a broad dynamic range within our measurement window (20 pW·µm^-2^ to 5 nW·µm^-2^). A minimum detectable power density of 20 pW·µm^-2^ is achieved, which we define as the point at which the signal equals the noise (signal-to-noise ratio, or SNR ≈ 1), calculated based on the incident, rather than the absorbed, LWIR light power. This performance is comparable to the single-channel detector performance reported previously^29^, and higher than other emerging methods such as ratiometric luminescence-based infrared detection^20^. The measurements were carried out with the available QCL with 9.5-µm wavelength, which is near the edge of the SiN_*x*_ absorption band; an output wavelength in the 11 ~ 12 µm range would be more favorable for optimal absorption and higher sensitivity down to the single-digit pW·µm^-2^ level—based on the absorption data shown in Fig. 1b. To evaluate the photo- and operational stability, the imager was continuously illuminated with LWIR radiation at a power density of 2.1 nW·µm^-2^ (*i.e*., 100 times the lowest detectable LWIR intensity) under ambient conditions for more than three days. As shown in Fig. 2c, the detected photothermal signal exhibits negligible degradation over the entire testing period, demonstrating the excellent stability of the (PEA)_2_PbI_4_/SiN_*x*_ bilayer LWIR imagers.

Although the large membrane-based imagers provide reasonable spatial resolution (Fig. 2a), lateral heat spreading across the (PEA)_2_PbI_4_/SiN_*x*_ membrane, as the dominant thermal dissipation pathway following each chopping cycle of the LWIR irradiation, inevitably leads to image blurring, as discernable in Fig. 2a. To mitigate this parasitic thermal crosstalk, we devised a unique strategy based on spatially structured probe illumination, as illustrated in Fig. 2d. Unlike the spatially uniform green probe used in the configuration shown in Fig. 1e, the probe light here is spatially filtered using a thin stainless-steel shadow mask positioned in its optical path. The mask, fabricated by laser-cutting, consists of periodic circular apertures, each with a diameter of 100 µm and a pitch of 200 µm. A lens placed before the mask ensures that the mask lies at the image plane of the probe beam, while a second imaging lens re-forms the patterned illumination onto the imager surface. A magnification ratio of 2:1 was achieved by proper selection of the lens pair, resulting in a disc diameter of 50 µm and a pitch of 100 µm on the imager plane. The resulting array of discrete, disc-shaped illumination spots acts as a matrix of independent “pixels”, each probing a spatially isolated region of the imager membrane. This structured probe configuration suppresses lateral heat diffusion between neighboring pixels, thus preserving spatial fidelity and improving imaging contrast of the LWIR photothermal signal. Using the structured probe illumination, we obtained a representative image of the “YALE CEE” logo as shown in Fig. 2e. A clear image can be observed, although the image quality is slightly affected by the film non-uniformity.

Since the aperture diameter and pitch size of the metallic mask can be further reduced, the spatial resolution achievable with this structured-probe scheme (Fig. 2d) can, in principle, be correspondingly enhanced. Aperture diameters down to 10 ~ 20 µm are readily achievable by high-quality laser cutting. However, lateral thermal diffusion following LWIR irradiation imposes a fundamental limit on the attainable spatial resolution. Rather than capturing the instantaneous spatial profile of the temperature rise induced by LWIR illumination, the continuous probe measures the time-averaged temperature distribution over the LWIR repetition interval ( ~ 20 ms, corresponding to a 50-Hz chopping rate). During this interval, lateral heat spreading smears the initially sharp illumination pattern, resulting in a blurred image on the imager.

To examine this intrinsic heat-diffusion-limited resolution, we performed finite-element method (FEM) simulations, in which the uniform, (PEA)_2_PbI_4_/SiN_*x*_ imager membrane is initialized with an impulsive square-wave-structured temperature jump at time zero, as schematically shown in Fig. 2f, representing illumination by a LWIR light with a fine line feature of width ${L}_{x}$. More details of the simulation can be found in Supplementary Note 1. It can be seen from the results in Fig. 2f that the initially abrupt edge becomes noticeably rounded due to lateral heat transfer, when averaged over the first 20 ms. A zoom-in cross-sectional view in Fig. 2g highlights this edge smearing. The line spread function (LSF) extracted from the blurred profile exhibits a Lorentzian-like shape, indicating the deviation of the temperature profile from the original sharp step. We quantify the degree of blurring by the ratio of the LSF’s full-width-at-half-maximum (FWHM) to the original feature width (L_x_). As shown in Fig. S4e, when the feature size is reduced to 25 µm, more than 20% edge blurring occurs due to thermal diffusion. This demonstrates that heat transfer within the membrane sets a fundamental resolution limit for the membrane-based imager when detecting fine LWIR spatial features.

**Fig. 3:**
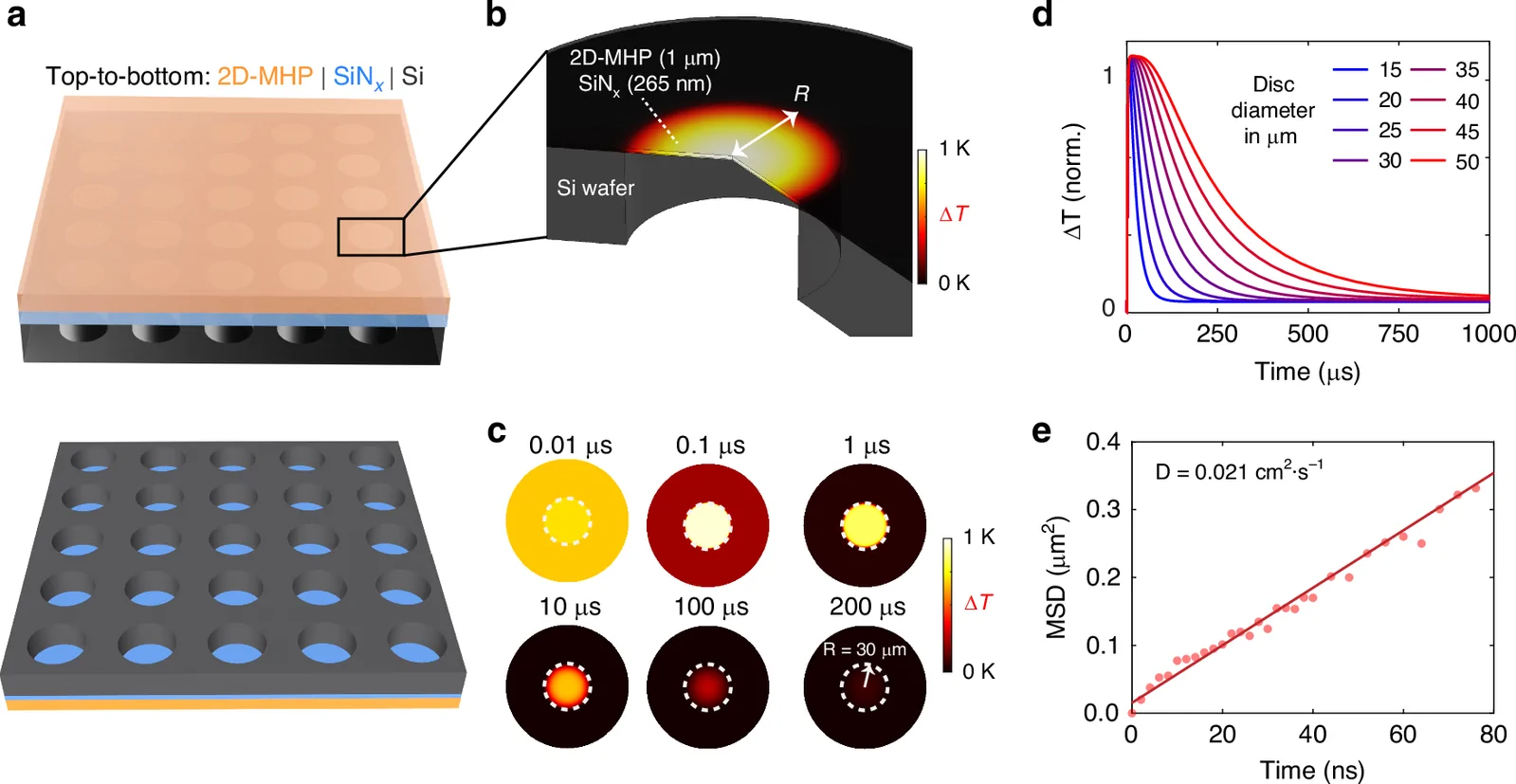
**a** Schematic drawing of the pixelated imager based on arrays of (PEA)_2_PbI_4_/SiN_*x*_ membrane windows supported by a Si wafer (top: top-to-bottom view; bottom: bottom-to-top view). **b** Simulated structure and temperature profile of an individual membrane pixel (at 10 µs delay time). **c** Temperature profiles at various delay times of the membrane disc with 60-µm diameter, assuming an impulsive temperature rise at time zero. **d** Simulated temperature decay of the center of the membrane disc as a function of disc diameter ranging from 15 µm to 50 µm. **e** Mean square displacement (MSD) of the diffusing heat with time measured by transient reflection microscopy on a SiN_x_ membrane

Recognizing the above limitation, we then developed a simple, yet effective imager architecture, targeting to completely eliminate pixel-to-pixel thermal crosstalk. The conceptual design is illustrated in Fig. 3a (top: top-down view; bottom: bottom-up view), where the continuous SiN_*x*_ membrane supported on a Si wafer is miniaturized and patterned into a periodic array of membrane pixels with sub-millimeter dimensions. In this design, the array periodicity is equivalent to the imager pitch size, while the ratio of the membrane diameter to the pitch determines the fill factor of the imager ( ~ 20% as determined by the membrane diameter of 50 µm and the pixel pitch of 100 µm). The pixelated imager structure can leverage the well-established Si processing infrastructure.

To quantify thermal isolation and to extract the characteristic time constants of temperature decay, we performed time-domain FEM simulation on an individual membrane pixel (Fig. 3b), assuming a uniform initial temperature rise within the SiN_*x*_ layer (i.e. LWIR light absorber). The thermal conductivities of (PEA)_2_PbI_4_, including both the cross-plane and the in-plane thermal conductivities, were taken from the literature^31–34^. The thermal conductivity of SiN_*x*_ was measured to be 5.1 W·m^-1^·K^-1^ using transient reflection microscopy, in which the thermal diffusivity (D) can be determined from the linear correlation between mean square displacement (MSD) of heat and time, i.e. ${{{\rm{MSD}}}}=2{Dt}$ (Fig. 3e), where t is the pump-probe delay time. More information about the thermal conductivity measurement of SiN_*x*_ is in Supplementary Note 2.

As shown in Fig. 3c, due to the high thermal conductivity of Si, and its large thickness (hence heat capacity) surrounding each membrane pixel, the geometry ensures complete thermal isolation between neighboring pixels, eliminating any pixel-to-pixel crosstalk. FEM simulation results for imager pixels with variable disc diameters, presented in Fig. 3d, reveal that the timescales of temperature decay within each pixel disc following impulsive LWIR thermal excitation at time zero reside in the sub- to single-ms regime.

**Fig. 4:**
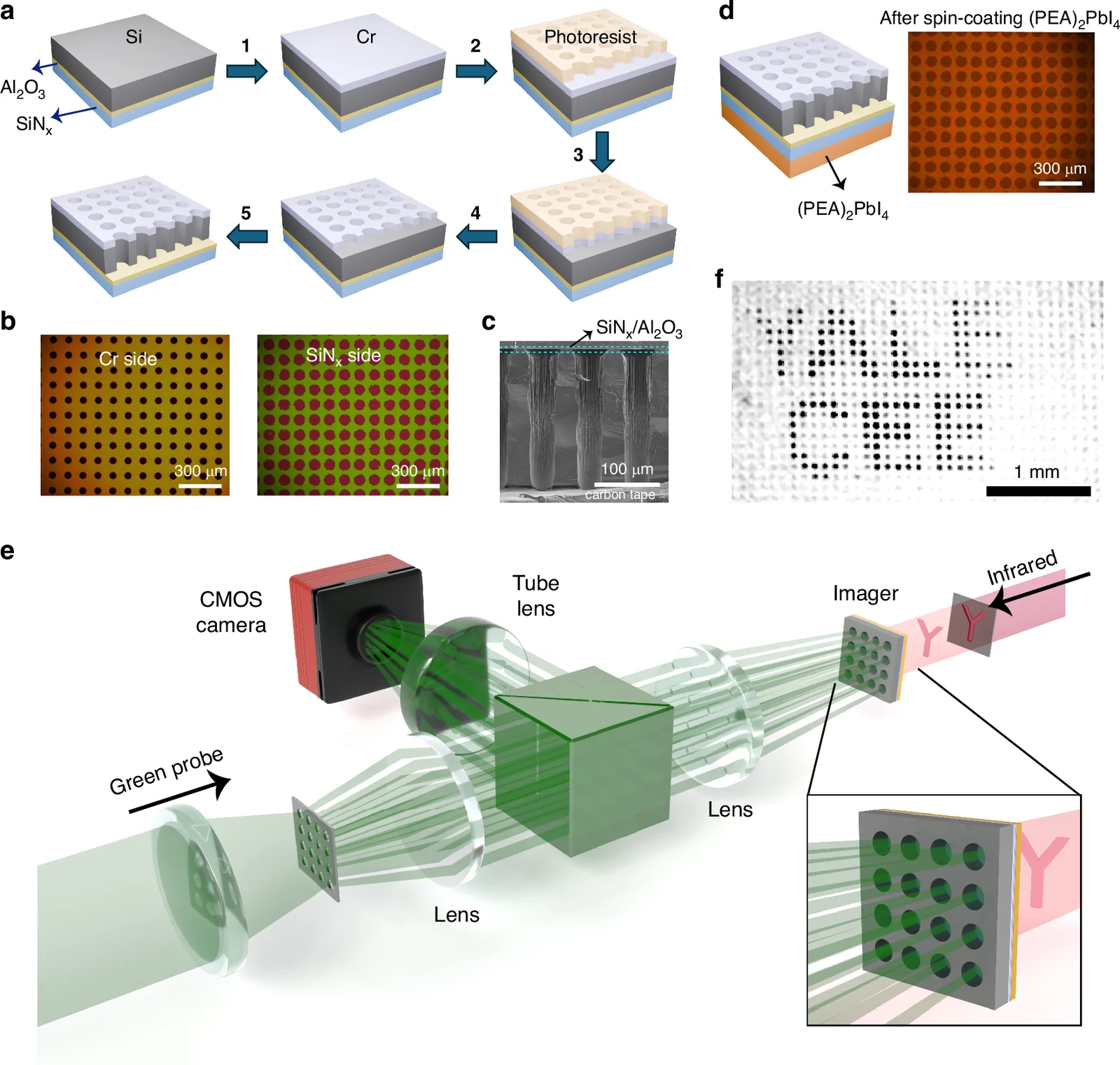
**a** Fabrication process of the periodic membranes (Step 1: thermal evaporation of chromium. Step 2: spin-coat photoresist, expose, and develop. Step 3: chromium wet etching. Step 4: removal of photoresist. Step 5: deep reactive-ion etching). **b** Optical micrographs of the finally obtained structure viewed from the Cr side and the SiN_*x*_ side. **c** Cross-sectional scanning electron microscopy (SEM) image showing through-Si-etching and the termination with thin Al_2_O_3_/SiN_*x*_ membrane. **d** Schematic illustration of the periodic-membrane substrate with a spin-coated layer of (PEA)_2_PbI_4_, forming the pixelated infrared imager, and an optical micrograph. The pixel pitch is 100 µm. **e** Schematic illustration of the detection scheme incorporating both structured visible probe beam and periodic membrane disc architecture for eliminating pixel-to-pixel crosstalk. **f** An image captured using the periodic membrane-based imager measured with the configuration in **e**

With this design concept, we fabricated periodic membrane-disc LWIR imagers. The multilayer stack, going from bottom to up, follows the configuration: Cr layer/Si wafer/Al_2_O_3_ film/SiN_*x*_ film/(PEA)_2_PbI_4_ film. In this architecture, the Cr layer serves as an etching mask for deep reactive-ion etching (DRIE) of Si, while the Al_2_O_3_ film is included as an effective etch-stop layer during the DRIE process. Figure 4a illustrates the overall fabrication flow. Fabrication begins with a commercially purchased Si/Al_2_O_3_ (120 nm)/SiN_*x*_ (290 nm) wafer, double-side polished, in which the Al_2_O_3_ and SiN_*x*_ layers are deposited on the top surface by thermal evaporation and plasma-enhanced chemical vapor deposition, respectively. The backside of the wafer is then coated with Cr by electron-beam evaporation and patterned via standard photolithography to define periodic holes in the photoresist. After selectively etching the exposed Cr through a wet-chemical process and removing the remaining photoresist, the Si wafer is covered with a hole-array Cr mask.

After this, Bosch DRIE is performed to fully etch through the 300 µm-thick Si substrate in the regions un-protected by the Cr mask, terminating on the Al_2_O_3_ layer on the opposite side. The final structure consists of a periodic array of Al_2_O_3_ (120 nm)/SiN_*x*_ (290 nm) membrane discs ( ~ 410 nm total thickness), separated by unetched Si regions that provide both mechanical rigidity and thermal isolation. This structured imager design ensures that each membrane disc behaves as an individual thermally isolated pixel, which enables high-fidelity, high-resolution photothermal imaging. Despite the large thickness ratio ( ~ 732:1) between the Si wafer (300 µm) and the final Al_2_O_3_/SiN_*x*_ membrane thickness ( ~ 410 nm total), we successfully achieved through-wafer etching, with optical micrographs taken from the Cr side and the SiN_*x*_ side shown in Fig. 4b. The process usually yields uniform films across inch-sized dimensions. The membrane discs appear slightly larger on the SiNₓ side due to lateral etching during the DRIE process. A scanning electron microscopy (SEM) cross-sectional image is shown in Fig. 4c, clearly demonstrating the flat, thin membrane on top. A spin-coating step is followed to deposit a (PEA)_2_PbI_4_ film on the sample, yielding the final structure of the pixelated LWIR imager, as illustrated in Fig. 4d.

We built a corresponding measurement setup, as illustrated in Fig. 4e, that synergistically combines the pixelated membrane-disc design and structured green sensing light illumination. The device design employs a pitch size of 100 µm and a disc diameter of 50 µm. Further increases in pixel density can be achieved by reducing the membrane-disc diameter, which would require a correspondingly thinner starting Si wafer to maintain high yield during the through-wafer DRIE. With this designed imager, we performed a LWIR detection experiment at a 50-Hz chopping rate. A representative image of the LWIR-illuminated “YALE CEE” logo, captured by the CMOS camera, is shown in Fig. 4f, demonstrating the spatial resolving power of the MIR imager at 9.5 µm. Importantly, the pixels produced by this design are sharply defined, and pixel-to-pixel crosstalk is effectively eliminated. Reduction in the pixel pitch down to below 20 µm may be achieved by optimizing the through-wafer DRIE process to yield higher etching aspect ratios. A smaller pitch and membrane diameter improve the mechanical stability of the membrane pixel, while still maintaining favorable thermal isolation due to the large thermal-conductivity and thermal-mass discrepancy between the 2D-MHP/SiN_*x*_/Al_2_O_3_ membrane and Si.

Notably, in this detection scheme, each disc-like probe-illumination spot must pass through the backside aperture of each membrane disc and reach the Al_2_O_3_/SiN_*x*_/(PEA)_2_PbI_4_ membrane stack (Fig. 4e, inset). A mismatch between the imager pitch (defined during photolithography) and the spacing of the structured probe spots (produced by the metallic mask) can accumulate across the field of view, ultimately resulting in misalignment between the probe pattern and the pixelated imager. To mitigate this effect and ensure precise spatial registration over centimeter-scale dimensions, we employed a lens pair with tunable magnification, which allows continuous adjustment of the probe pattern magnification to achieve accurate alignment. The shadow mask was mounted on a translation and rotation stage, allowing alignment optimization. Supplementary Fig. S7 illustrates the initial alignment of the structured probe relative to the periodic membrane-disc array. The current measurements employ a cost-effective LED as the probe source, with a spectral bandwidth of 10 nm defined by a bandpass filter. Alternatively—although not explored in this study—a continuous-wave laser could be used in place of the LED, particularly given the low cost of lasers in the green wavelength range. By properly selecting the probe-laser wavelength to coincide with the excitonic resonance, the probe can be better matched to the wavelength at which the TCR is maximized^29^. In addition, a microlens array or a spatial light modulator may replace the metallic mask to generate periodic structured-probe illumination, offering a more compact and potentially more efficient measurement architecture.

## Discussion

In conclusion, we demonstrate that 2D-MHPs—exemplified by (PEA)_2_PbI_4_—enable all-optical detection of LWIR light near the 10-µm wavelength regime with desirable imaging performance. This photothermal detection strategy leverages the large exciton-resonance–enhanced TCR inherent to these strongly excitonic, cost-effective, and solution-processable layered materials. In principle, other Ruddlesden-Popper and Dion-Jacobson phase 2D-MHPs beyond (PEA)_2_PbI_4_ can also be used for LWIR detection due to their low thermal conductivity and high TCR, while pulsed laser deposition and thermal evaporation may be employed as alternatives to solution processing to enable improved process repeatability. Flexible and stretchable substrates with sub-µm thickness can be employed to replace the SiN_*x*_ to enable deformable devices. A direct comparison of standardized detector performance metrics, such as noise-equivalent power (NEP) and specific detectivity (${D}^{* }$), is not straightforward, as our platform operates based on an all-optical detection principle that differs from conventional IR detectors with electrical readout (e.g., VOₓ and α-Si microbolometers). At the material level, the PEA-based system exhibits a figure of merit (TCR/(κ·Cₚ)) approximately one order of magnitude better than VOₓ^29^, indicating an intrinsic advantage.

The demonstrated, proof-of-concept all-optical imaging of LWIR light currently requires components such as a chopper, structured-illumination and imaging optics for the LED probe, which increases system complexity. In future implementations, this can be alleviated by using compact green laser sources, microlens arrays, and electronic shutters to enable smaller device footprints. While our proof-of-concept demonstration is based on (PEA)_2_PbI_4_, we anticipate that other 2D-MHPs, or layered metal-organic chalcogenides and 2D transition metal dichalcogenides, which exhibit exceptional structural and chemical tunability, may offer comparable or improved TCR values for all-optical LWIR detection^35–38^. By incorporating appropriate solution-processable light-absorbing materials, including transparent conducting oxides^39^, correlated materials^40^, graphene and *h*-BN^41,42^, conducting polymers^43^, as well as carbon nanotubes and conjugated polymers^44–46^, the imaging modality can be extended to longer wavelengths spanning the far-infrared to the terahertz and sub-terahertz regimes. The fundamental insights revealed here may enable detectors for real-time tracking of thermal radiation, rapid and accurate molecular and viral identification, and high-resolution, high-SNR imaging for scientific instrumentation. Moreover, the all-optical detection architecture may support remote sensing, allowing signals to be interrogated non-invasively by external optical probes.

## Materials and methods

### Grazing-incidence wide-angle X-ray scattering (GIWAXS)

Grazing-Incidence Wide-Angle X-ray Scattering (GIWAXS) measurements were performed at the Soft Matter Interfaces (SMI) beamline (12-ID) of the National Synchrotron Light Source II (NSLS-II) at Brookhaven National Laboratory. The experiment utilized a monochromatic X-ray beam with an energy of 16.1 keV (corresponding wavelength $\lambda \approx 0.77{{{\mathrm{\AA}}}}$) and an energy resolution of $\triangle E/E=0.01 \%$. The beam was focused to dimensions of 200 µm (horizontal) × 30 µm (vertical) at the sample position. Scattered X-rays were detected using a PILATUS3 900 kW detector (Dectris, Switzerland), featuring a 1475 × 619 array of 0.172 mm × 0.172 mm pixels, positioned at a sample-to-detector distance (SDD) of 275 mm.

Measurement procedure: thin film samples, approximately 1 cm × 1 cm in size, were mounted on a high-precision piezo-motorized stage. GIWAXS patterns were collected at multiple incident angles (${\alpha }_{i}$) relative to the substrate plane, typically including 0.05, 0.10, 0.15, and 0.30 degrees, to probe different depths within the films. For statistical robustness, measurements were taken at three distinct positions on each sample, with an exposure time of 1 second per position. To access a wide range of scattering vectors (q), the detector was rotated along an arc, and the resulting series of 2D diffraction patterns were computationally stitched together using custom software. Calibration of the scattering angle (2θ) and subsequent q-space conversion was performed using a silver behenate (AgBH) powder standard (${d}_{001}=58.38{{{\mathrm{\AA}}}}$).

### Pump-probe transient reflectance

Femtosecond (fs) MIR pump pulses were generated using an optical parametric amplifier (OPA, Orpheus-One-HE) and focused onto the sample with a CaF_2_ lens with 250-mm focal length, with a spot size of ~150 µm. The OPA was powered by a femtosecond laser amplifier (Pharos) operating at 2 kHz repetition rate with 170 fs pulse width, delivering 0.9 mJ pulse energy to the OPA. Nanosecond (ns) probe pulses were produced by a fiber-coupled supercontinuum ns laser (Leukos, Disco) and time-delayed using a digital delay generator (SRS DG645). The probe laser output was spectrally defined using appropriate long-pass and short-pass filters, enabling broadband measurements around the exciton resonance of each 2D-MHP. An objective with 5X magnification and 0.14 NA was used to focus the probe onto the sample with a spot size of ~5 µm. VPVP spectroscopic detection was enabled by a USB spectrometer (AvaSpec-ULS2048CL-EVO).

### Large-membrane LWIR imager fabrication

A large SiNx/Si membrane (lateral size of 1.5-2 cm) was fabricated by wet etching using a 32 wt% KOH solution containing isopropanol (IPA). The etchant was prepared by mixing 38.4 mL of 45 wt% KOH solution with 22.8 mL of DI water, followed by the addition of 6 mL of IPA to reduce the surface roughness of the etched samples. A large pinhole was created on the backside of a commercially customized Si wafer (coated with 265 nm-thick SiNx on both sides) using a diamond scribe to expose the Si layer to the etchant. After the SiNx/Si wafers were immersed in the prepared solution, the mixture was heated to 65 °C and the wafers were etched for 24 hours.

The (PEA)_2_PbI_4_ precursor solution was prepared by dissolving 0.5 mmol of lead(II) iodide and 1.0 mmol of phenethylammonium iodide in 0.5 mL of N,N-dimethylformamide (DMF). The etched SiNx/Si wafers were treated with air plasma for 10 min to improve surface wettability. The (PEA)_2_PbI_4_ films were then deposited by spin-coating 80 μL of the precursor solution onto preheated (130 °C) SiNx/Si substrates at 3000 rpm for 30 s with an acceleration rate of 1000 rpm/s, followed by annealing at 100 °C for 5 min.

### Pixelated LWIR imager fabrication

A commercially customized double-side-polished silicon wafer was used, with one side coated with a 120 nm Al_2_O_3_ layer (serving as the etch-stop layer) and a 290 nm SiNx layer.

1. Chromium deposition: The wafer was cleaned with O_2_ plasma using an AutoGlow 200 Etcher (Glow Research) for 5 minutes. Subsequently, a 100 nm chromium layer was deposited on the Si side using a UHV Electron Beam Evaporator (PVD Products).
2. Photolithography: The pixel pattern was defined by photolithography. The substrate was first dehydrated and primed in a YES HMDS Prime Oven for 25 minutes to improve photoresist adhesion. A positive photoresist (Shipley S1805) was then spin-coated at 4000 rpm for 60 s and baked at 105 °C for 1 minute. The photoresist was subsequently exposed using an MLA 150 Maskless Aligner (Heidelberg Instruments), developed in MF-319 developer for 30 seconds, rinsed with deionized water and dried with an air blow gun.
3. Chromium etching: The exposed chromium was wet etched for 90 seconds using a chromium etchant to form the etching mask, followed by rinsing with DI water and drying with an air gun.
4. Photoresist removal: The residual photoresist was stripped using N-methyl-2-pyrrolidone (NMP) at 50 °C for 1 minute, then rinsed with DI water and dried.
5. Si-wafer etching: The wafer was completely etched through to form the multi-pixel membrane by deep reactive-ion etching (DRIE) using the Bosch process on an Oxford Plasmalab 100 system. Prior to etching, the wafer was cleaned with O_2_ plasma for 1 minute using an AutoGlow 200 Etcher (Glow Research).
6. Active layer coating: The perovskite layer was spin-coated onto the SiNx side using the same procedure as that for the large-membrane LWIR imager.

## Supplementary information

Supplementary Materials

## Data Availability

Data sets generated during the current study are available from the corresponding author on reasonable request.
