# Supplementary material for: All-optical 2D perovskite photodetector arrays for pixelated long-wave infrared imaging
Source: Light Sci Appl. 2026 Nov 2;15:393. doi: 10.1038/s41377-026-02442-y (PMC13724749; doi:10.1038/s41377-026-02442-y)
Supplement: Supplementary file 1 [file 41377_2026_2442_MOESM1_ESM.pdf]

Supplementary Information for

All-optical 2D perovskite photodetector arrays for pixelated long-wave infrared imaging

Yanyan Li[1,2], Zheng Qian[1,2], Du Chen[1,2], Conrad A. Kocoj[1,2], Shunran Li[1,2], Renee Wu[1,2], Xiang Shi[1,2], Ralu Divan[3], David A. Czaplewski[3], Benjamin T. Diroll[3], Ming Lu[4], Yugang Zhang[4], Shu Hu[1,2], Peijun Guo[1,2,*]

[1]Department of Chemical and Environmental Engineering, Yale University, New Haven, CT 06520, USA

[2]Energy Sciences Institute, Yale University, West Haven, CT 06516, USA

[3]Center for Nanoscale Materials, Argonne National Laboratory, Lemont, IL 60439, USA

[4]Center for Functional Nanomaterials, Brookhaven National Laboratory, Upton, NY 11973, USA

Supplementary Note 1. Finite-element thermal simulation of the $(PEA)_2PbI_4/SiN_x$ imager

1. Simulation of diffusion-limited spatial resolution of single-channel imager

Finite-element simulation with COMSOL Multiphysics was conducted using the actual geometry of the single-channel $(PEA)_2PbI_4/SiN_x$ imager as shown in **Fig. S4a**. For simplicity, we assumed a one-dimensional square-wave feature of the incident LWIR to be imaged, thus simplifying the scenario to a two-dimensional model representing the cross-sectional view of the imager as shown in **Fig. S4b**. The square-wave initial temperature of the SiN_x layer represents an instantaneous spatial temperature profile of the imager after LWIR incidence. The probe light is incident from the SiN_x side onto $(PEA)_2PbI_4$, sampling the temperature of the $(PEA)_2PbI_4$ layer with a depth of 100 nm (the penetration depth of the 510-nm probe light). In the working condition of our imager, the probe is measured at 100 Hz – corresponding to a repetition period of 10 ms – therefore the imager essentially captures the temperature profile averaged temporally over 10 ms (**Fig. S4c**) and spatially over a depth of 100 nm (**Fig. S4d**) after LWIR illumination. The simulated temperature spatial profile averaged for 10 ms is presented in **Figs. S4c and S4d**, which shows a noticeable blurring at the edge of the initial square-wave feature. The line spread function (LSF) of the blurred edge can be calculated as the derivative of the profile, which shows a Lorentzian-like shape as shown in **Fig. 2g**. The FWHM of the fitted Lorentzian can be used as a figure of merit quantifying the extent of blurring due to thermal diffusion. We define the percentage blurring as FWHM/L_x, which increases with smaller feature size (L_x), indicating a larger deviation between the image captured by the imager and actual LWIR profile. As shown in **Fig. S4e**, FWHM/L_x decreases exponentially with L_x, approaching 10% around L_x = 50 μm. The fact that L_x here is orders of magnitude larger than the probe wavelength we are using to form image implies that this is not subject to the diffraction-limit but rather a thermal-diffusion-limited spatial resolution. In the membrane-based LWIR imager proposed here, thermal diffusion between neighboring pixels thus potentially imposes a fundamental limit to the spatial resolution of the imager.

2. Simulation of an individual pixel of the multi-channel imager

Due to the symmetry of the imager pixel, an azimuthally symmetric simulation was performed with COMSOL Multiphysics, in which the geometry is taken from the fabricated imager while the hole radius (R) is swept from 15 to 50 μm as shown in **Fig. S5a**. An initially uniform temperature rise (10 K) of the SiN_x layer is assumed to mimic a uniform illumination of LWIR on the imager. The subsequent thermal dissipation towards the adjacent layers, *i.e.*, $(PEA)_2PbI_4$ and Al_2O_3, and eventually Si occurs with a spatially dependent rate. In regions supported by Si (away from the pixel center), heat dissipates much faster while persisting in the pixel region for up to several tens of microseconds (**Fig. S5b**).

The simulation parameters, including the materials properties and interface thermal conductance used in the model are summarized in **Table S1**.

**Materials properties**	$(PEA)_2PbI_4$	SiN_x	Al_2O_3	Si
Mass density ($kg \cdot m^{-3}$)	2464.6	3170	3990	2330
Heat capacity ($J \cdot kg^{-1} \cdot K^{-1}$)	457.4	760	719	710
Thermal conductivity ($W \cdot m^{-1} \cdot K^{-1}$)	$\begin{bmatrix} 0.15 & 0 & 0 \\ 0 & 0.15 & 0 \\ 0 & 0 & 0.3 \end{bmatrix}$	5.1	37	142.2
Interface property	$(PEA)_2PbI_4/SiN_x$	SiN_x/Al_2O_3	Al_2O_3/Si	
Interface thermal conductance ($MW \cdot m^{-2} \cdot K^{-1}$)	80	200	200	

Table S1 Material and interface properties used in the FEM simulation.

Supplementary Note 2. Thermal conductivity measurement of the SiN_x film

The transient reflection microscopy is schematically illustrated in **Fig. S6a**, where the OPA – powered by a femtosecond laser amplifier (Pharos) operating at 2 kHz repetition rate and 170-fs pulse width – outputs a 640-nm pump light. The pump beam is spatially filtered by a 25-µm pinhole and expanded by three times before being focused onto the sample by a 50X objective. The nanosecond (ns) probe light is generated by a fiber-coupled supercontinuum ns laser (Leukos, Disco) and time-delayed using a digital delay generator (SRS DG645). The probe laser is then steered into a monochromator to produce a narrow-bandwidth (~8 nm in full-width-half-maximum) output centered at 550 nm. A wide-field lens (WFL) with 400-mm focal length then focuses the probe light at the back aperture of the objective to enable wide-field illumination. The reflected probe light is then focused on a CMOS camera (Pixelink-PL-D753) by a 400-mm tube lens.

The heat generated by the focused pump changes the local refractive index of SiNx, inducing spatial-dependent changes in the reflection of the probe, i.e., $\Delta R/R_0$, where R_0 is the initial reflectivity. Such changes are captured by the CMOS camera as shown in **Fig. S6b**, where the red shades indicate increase in the reflection. The expansion of the contrast with time is the result of heat diffusion. Due to the isotropic nature of SiN_x, we performed an azimuthal averaging around the signal center to produce a spatiotemporal distribution of the diffusing heat shown in **Fig. S6c**. The distribution at each time can be fitted by a Gaussian with a standard deviation of $\sigma(t)$ as presented in **Fig. S6d**. The mean square displacement (MSD), defined as $\text{MSD} = \sigma(t)^2 - \sigma(0)^2$, has a linear relationship with time for normal heat diffusion, which is $\text{MSD} = 2Dt$. Here D is the thermal diffusivity, and can be transformed to thermal conductivity by $\kappa = D\rho C$, where ρ and C are the mass density and heat capacity of SiN_x, respectively (**Table S1**). The thermal diffusivity of

0.021 cm^2·s^{-1} extracted from MSD as shown in **Fig. 3e** then indicates a thermal conductivity of 5.1 W·m^{-1}·K^{-1}, well aligned with the typical values for PECVD-grown SiN_x.

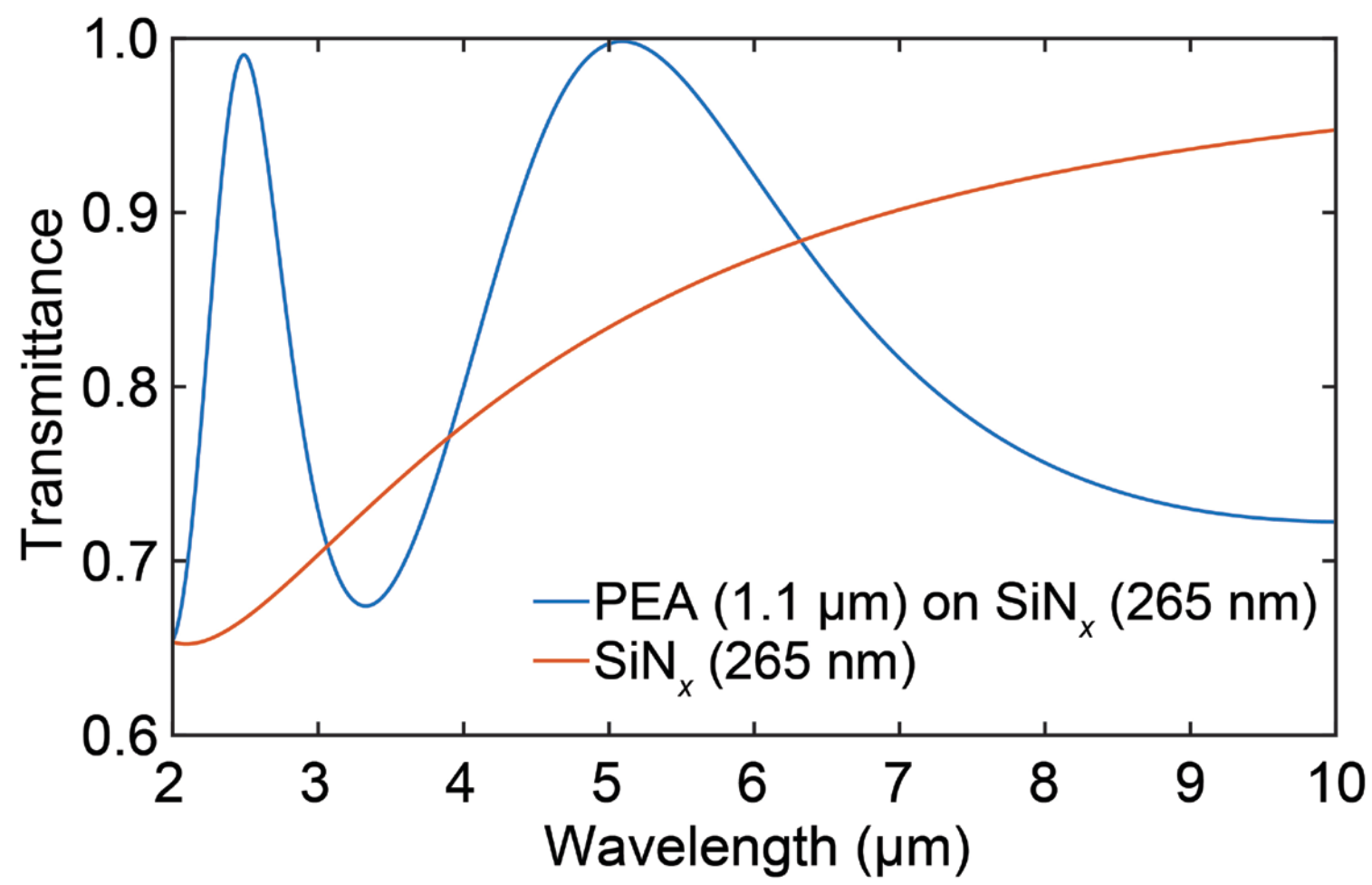

Fig. S1 Transfer-matrix-calculated transmittance spectra for a bare SiN_x membrane (265-nm thick) and a bilayer stack of $(PEA)_2PbI_4/SiN_x$ (1.1 μm/265 nm), showing modulation of transmittance by thin-film interference effect. A constant refractive index of 1.8 and 2.0 are assumed for $(PEA)_2PbI_4$ and SiN_x, respectively. The vibrational absorption of SiN_x is ignored in the transfer matrix calculation to isolate the interference effects.

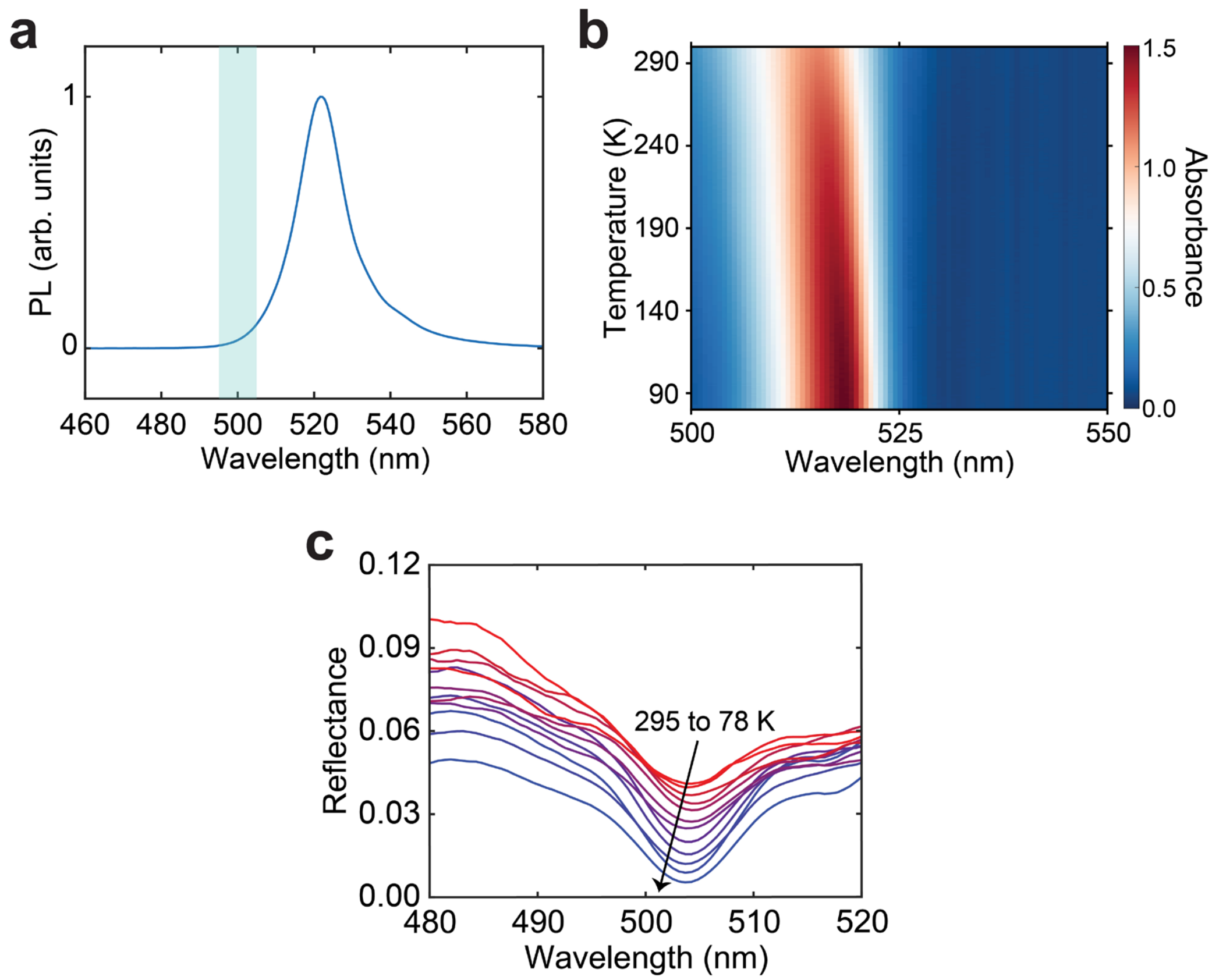

Fig. S2 **a** Photoluminescence (PL) spectrum of spin-coated $(PEA)_2PbI_4$ film on a SiN_x membrane at room temperature. **b** Absorbance, and **c** Reflectance spectra of spin-coated $(PEA)_2PbI_4$ film on glass substrate as a function of temperature.

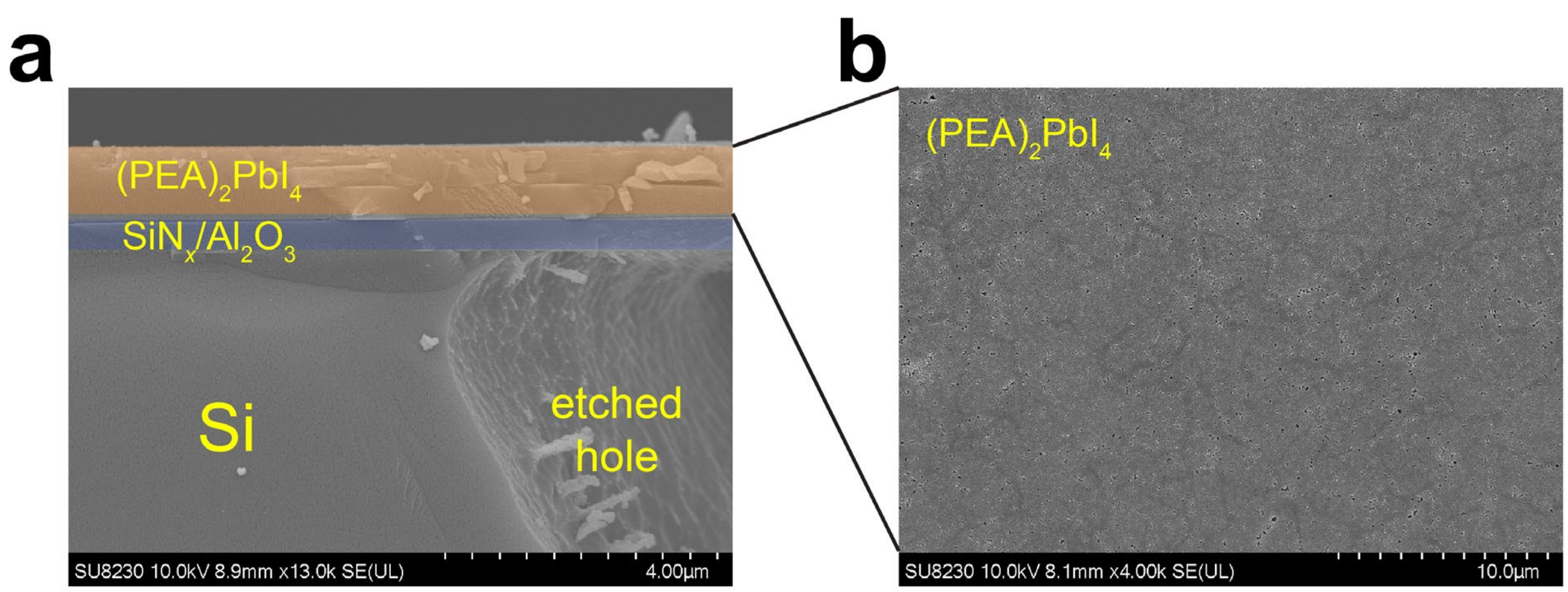

Fig. S3 **a** Cross-sectional SEM image showing spin-coated $(PEA)_2PbI_4$ layer on top of the SiN_x/Al_2O_3 membrane at the intersection of unetched and etched Si areas. **b** The corresponding SEM image of the $(PEA)_2PbI_4$ layer.

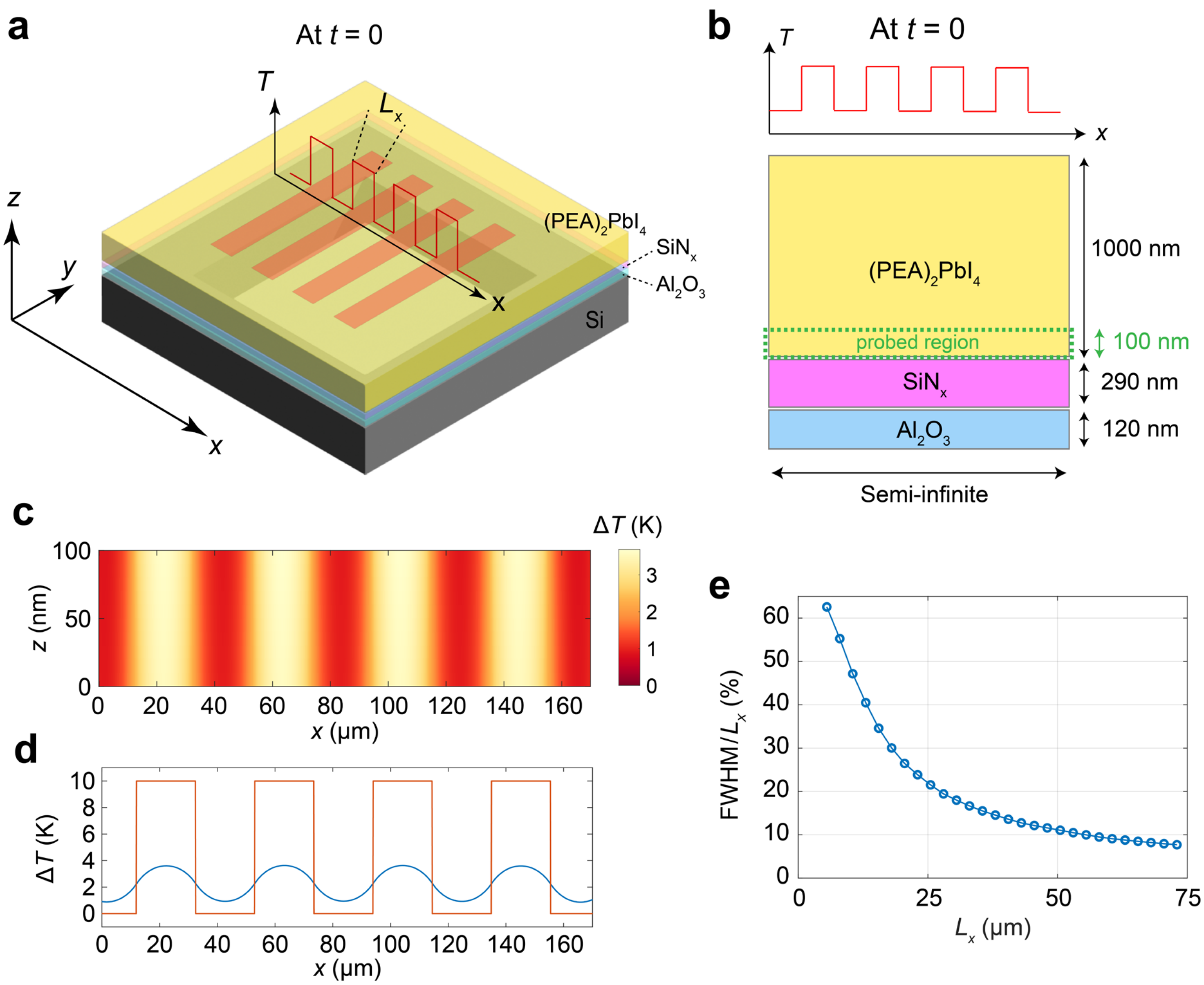

Fig. S4 Simulation of diffusion-limited spatial resolution of single-channel LWIR imager. **a** Initial setup of the simulation featuring a square-wave initial temperature profile representing the spatial profile of the incident LWIR. **b** Schematics of the simplified 2D simulation on the cross-section of the imager. **c** The probed temperature profile (up to a depth of 100 nm) averaged over the first 10 ms when the feature size (L_x) is taken to be 20 μm. **d** The initial and 10-ms temperature profile averaged over the probe depth from panel **c**. **e** The percentage of edge blurring (FWHM/L_x) under different feature size (L_x).

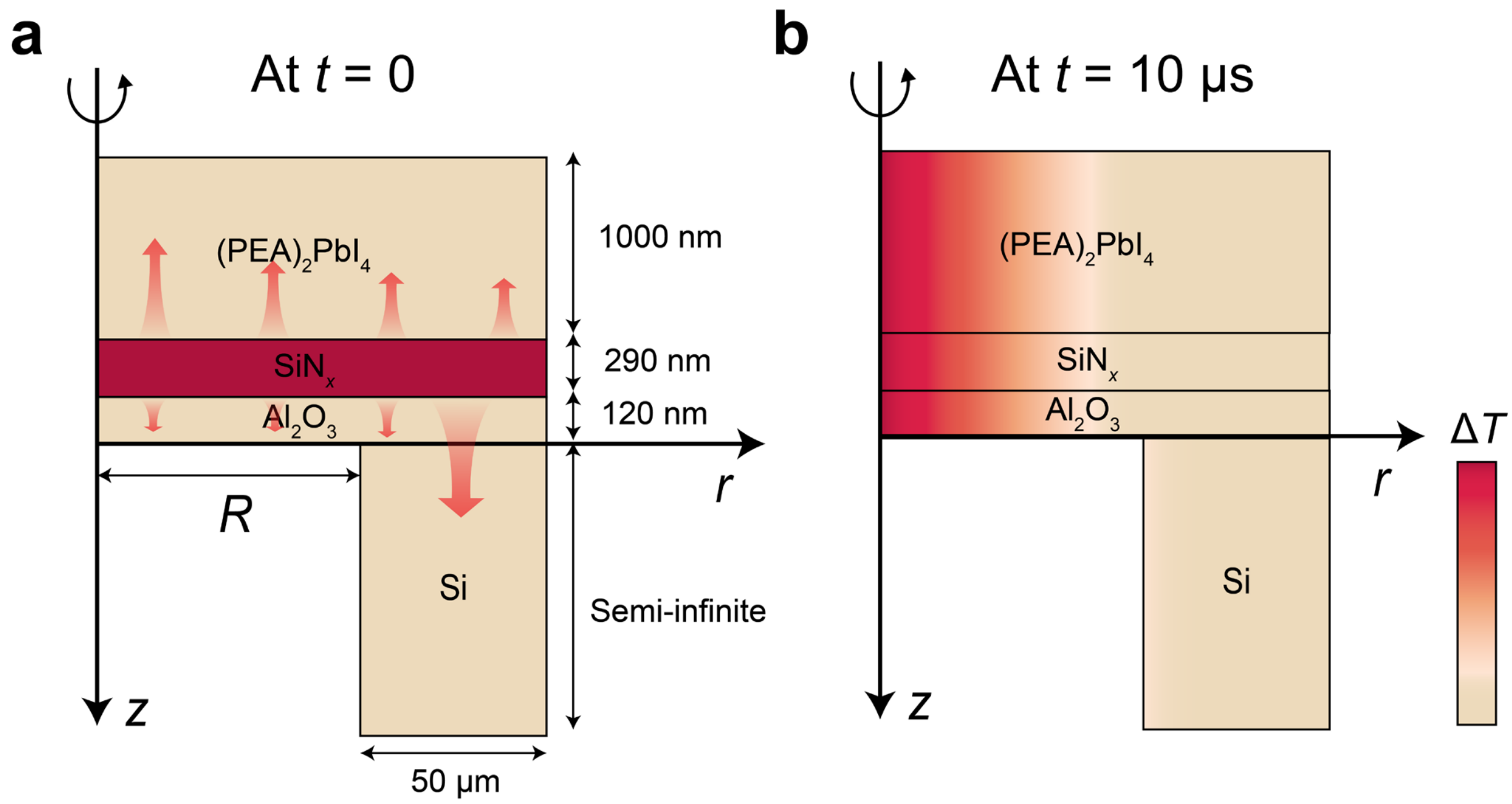

Fig. S5 Simulation of an individual pixel in the multi-channel imager. **a** Geometry and initial temperature profile. **b** The temperature profile of the pixel after 10 μs.

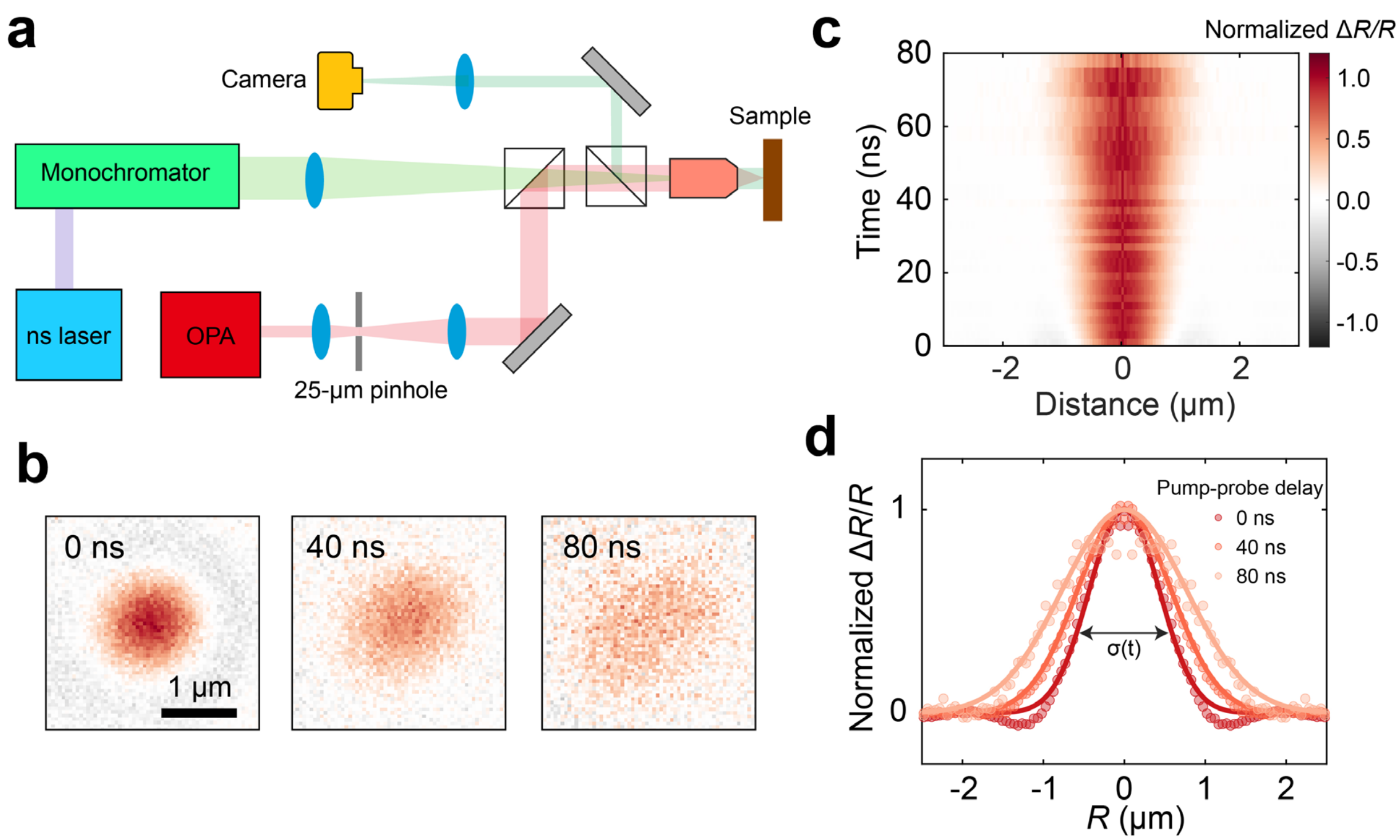

Fig. S6 Transient reflection microscopy on SiN_x. **a** Schematics of the experimental setup. **b** Snapshots of the heat diffusion. **c** Azimuthal-averaged spatiotemporal map of heat. Note that the profile is mirror imaged at zero distance. **d** Measured heat distribution profile fitted by a Gaussian distribution with standard deviation of $\sigma(t)$ at time t.

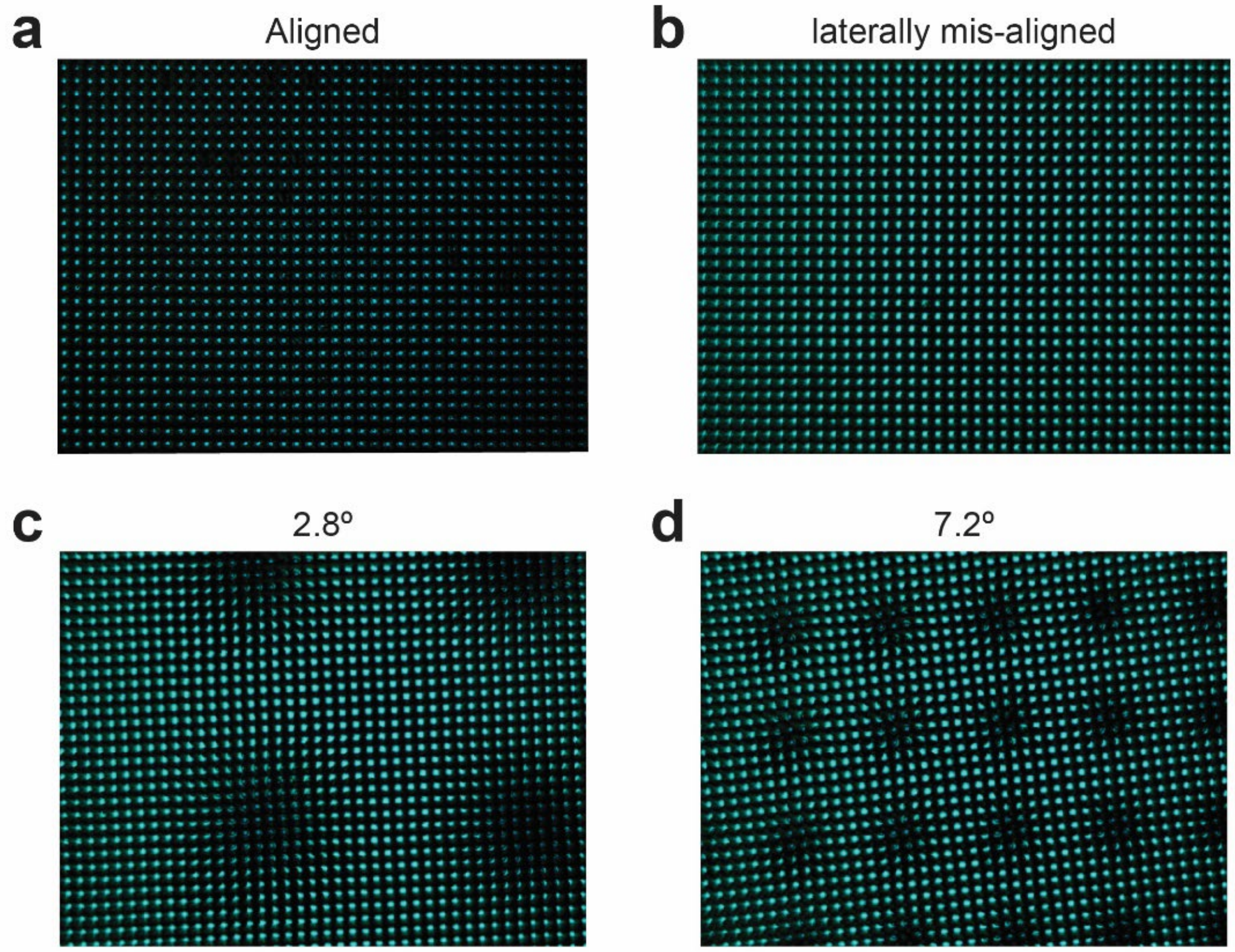

Fig. S7 Optical micrographs showing the alignment of the structured probe relative to the periodic membrane-disc array. **a** Structured probe aligned to the membrane discs containing intentionally partially broken holes. **b** Structured probe misaligned from the membrane-disc array laterally (hence the reflection is completely from the Si substrate). **c** Structured probe misaligned from the membrane-disc array by 2.8º. **d** Structured probe misaligned from the membrane-disc array by 7.2º.
